# Don’t tell me, show me: Reactions from those with lived experience to the 2020 revised IASP definition of pain

**DOI:** 10.1002/pne2.12059

**Published:** 2021-07-18

**Authors:** Isabel Jordan, Rachel Martens, Kathryn A. Birnie

**Affiliations:** ^1^ Squamish BC Canada; ^2^ Calgary AB Canada; ^3^ Department of Anesthesiology, Perioperative, and Pain Medicine Department of Community Health Sciences Cumming School of Medicine University of Calgary Calgary AB Canada; ^4^ Alberta Children’s Hospital Research Institute University of Calgary Calgary AB Canada

**Keywords:** lived experience, pain, pain definition, pain experience, parents, pediatric

## Abstract

In 2020, the International Association for the Study of Pain (IASP) revised the definition of pain, providing an update to IASP’s original definition in place since 1979. The origins of the field of pediatric pain, born in part in the 1980s from the advocacy of Jill Lawson, mother to Jeffrey Lawson who experienced gross inadequacies in pain care as a premature infant, emphasize the critical need to listen to and act with the voice of people living with pain and their families and caregivers. Furthermore, the field of pediatric pain emerged in the mid‐1980s marking this revised definition of pain as the first opportunity within which the experience and science of pain in childhood has been considered. This invited commentary offers two lived experience reactions to the revised IASP definition of pain, from the perspective of one's own experience with pain during childhood and as parents and family members. Together, they highlight that the value of a revised definition must be judged on its ability to directly benefit children experiencing pain and their families. Their skeptical hope reflects their lived experience backed by empirical evidence demonstrating continued inequities and inadequacies in preventing and managing pediatric pain. We must mobilize together to ensure change in culture, knowledge, and behavior. With the combined efforts of researchers, healthcare professionals, and policymakers, in partnership with diverse people with lived experience, we can ensure that more effective action is taken to rapidly improve pain for children and their families.

## 1 INTRODUCTION

In 2020, the leading international professional pain organization, known as the International Association for the Study of Pain (IASP), updated their longstanding definition of pain that had been in use since 1979.^8^ The revised definition describes pain as “*an unpleasant sensory and emotional experience associated with, or resembling that associated with, actual or potential tissue damage*.”^8^ The process to revise the definition of pain took two years and was overseen by an international IASP Presidential Task Force consisting of 14 individual experts in clinical and basic pain science, and consultative input from IASP membership and the public.^8^ The Task Force used multiple approaches to develop a revised definition of pain, including a modified Delphi survey, meetings (web conferences, face‐to‐face), email discussions, and qualitative content analysis of public review comments.^8^

Also important are the notes that accompany the revised definition of pain that highlight that:

● Pain is always a personal experience that is influenced to varying degrees by biological, psychological, and social factors.
● Pain and nociception are different phenomena. Pain cannot be inferred solely from activity in sensory neurons.
● Through their life experiences, individuals learn the concept of pain.
● A person's report of an experience as pain should be respected.
● Although pain usually serves an adaptive role, it may have adverse effects on function and social and psychological well‐being.
● Verbal description is only one of several behaviors to express pain; inability to communicate does not negate the possibility that a human or a nonhuman animal experiences pain.^8^

By its very nature, pain is subjective. Both the 1979 definition and revised 2020 IASP definition of pain highlight this in the accompanying notes. Historical conceptualizations of pain have further emphasized that point with Margo McCaffery in 1968 writing, “*Pain is whatever the experiencing person says it is, existing whenever he says it does*.”^6^ Given this, it is important to consider the perceptions of people with lived experience to this revised definition of pain. Some effort to seek and integrate the voice of people with lived experience of pain was done as part of the IASP Presidential Task Force's efforts consultation process, including 339 individuals (or 42% of all consulted) identifying as an individual living with pain, an individual with pain‐related disability, or a care provider for a person living with pain. However, it is unknown how many of those individuals have experience with pain during childhood themselves or as parents or caregivers of children with pain. Furthermore, no person with lived experience was included in the Task Force.

Increasingly, pediatric pain research and care have recognized the expertise of people with lived experience (children, parents/caregivers), involving them as collaborators in design and mobilization of research, as well as equal partners in developing and carrying out effective pain management plans. A 2020 Lancet Child and Adolescent Health Commission on pediatric pain critically identified that transformative change will only occur through cross‐sector collaboration between researchers, healthcare professionals, policymakers, funders, and patients and families.^3^ In 2020 IASP formed the Global Alliance for Partners for Pain Advocacy (GAPPA), Task Force intended to champion the lived experience voice and perspective in IASP governance structure, membership models, special interest groups, organization chapters, organizational strategy, and programs.^1^, ^4^

The revolutionary origin of the field of pediatric pain was born in the mid‐1980s by a mother with lived experience highlighting gross inadequacies in pain care, coupled with public outcry regarding the ethics of emerging science.^7^ Jill Lawson advocated widely for improved pediatric pain care in the public media and in scientific journals, including a letter she published in the journal *Birth* detailing the cardiac surgery performed on her prematurely born son, Jeffrey Lawson, while he was conscious, given only paralytics so as not to move, and without anesthetic.^5^ Jeffrey died 5 weeks later on March 31, 1985, after he suffered shock and multiple organ failure. What follows from this time is exponential growth in scientific study and improved practices focused on pain in infancy, childhood, and adolescence.^2^, ^3^ This history demonstrates the value of listening to lived experience in creating revolutionary change in perspective and practice, and the potential continuation of dangerous practice if lived experience is ignored. The development of the field of pediatric pain from the mid‐1980s, after IASP’s initial 1979 definition of pain was written, marks this 2020 revised definition of pain as the first opportunity within which the experience and science of pain in childhood has been considered.

In this spirit of empowering lived experience perspectives, what follows are two first‐person responses to the revised definition of pain as it pertains to pain during childhood, followed by concluding remarks that highlight key messages and implications.

## 2 LIVED EXPERIENCE PERSPECTIVES

Isabel Jordan (she/her) is a patient partner and lives in Squamish, British Columbia, Canada. In the spirit of reconciliation, Isabel is grateful to live, work, and play in the traditional, unceded territory of the Sḵwx̱wú7mes First Nation.

In my teenage years in the late 1980s I began to experience pain and disability. Almost overnight, I went from a high performance athlete and academic overachiever to somebody who didn't understand why her own body was betraying her. Pain became my daily companion and getting help was no easy task. While my own family showed unwavering support despite my mysterious symptoms, it was much more difficult over the ensuing years to get understanding from healthcare providers, educators, peers, and the support systems I needed in society. Not only was my pain invisible, but as we couldn't find a mechanical source for the pain, a diagnosis we could point to, indeed an ‘associated actual or potential tissue damage’ (what was then the IASP definition of pain^8^), the longer I had pain without a diagnosis, the greater stigma and the less support I received. This was a long time ago. Time, as they say, marched on. One hoped things have changed for the better in a system that purports to value patient centered care, shared decision making, and patient partnership.

In the past few years, I have had incredible opportunities to work within the pain research community as a parent partner. I have come to understand how far the thinking has come within this research and clinical community and was heartened to see the new definition of pain come from the IASP. There is one phrase that is different, one word, that can create a sea change in care for those who experience pain. The addition of the phrase ‘resembling that associated with’ adds so much to the definition of pain because it highlights the subjective experience of pain in the definition. It creates space for autonomy, for a lack of diagnosis, for the fact that pain, in and of itself, is a diagnosis and needs to be treated, whether or not it is associated with a separate diagnosis with ‘actual or potential tissue damage’. After a heady moment of excitement at this change, I thought, so what? What happens next?

My experience with pain didn't end with me. I parent two children who have experienced pain, chronic and acute. They were not able to access pain care through specialized pain services until well into their ‘pain journey’ in their later teenage years. The healthcare professionals they have seen are likely working from old models and an outdated definition of pain and treat them with those biases and that antiquated knowledge. And that causes harm. While I applaud the change of definition, I want to see it where it does the most good, where most will seek care for the pain, and that isn't with pain specialties. It is with family practitioners, pediatricians, emergency department doctors, nurses, and others. I want to know what's next. How do we take this knowledge and intentionally engage outside of the pain community so that others can improve their practice when it comes to pain care. This is knowledge that needs to spread. So, we have a new definition for pain. What's next?

Rachel Martens is a patient partner from Calgary, Alberta, Canada. In the spirit of reconciliation, Rachel acknowledges the traditional territories of the Blackfoot Confederacy (Siksika, Kainai, Piikani), the Tsuut’ina, the Îyâxe Nakoda Nations, the Métis Nation (Region 3), and all people who make their homes in the Treaty 7 region of Southern Alberta.

My relationship with pain meant supporting two people in my life whose experiences, while unique in presentation, bore some similarities in how their pain was recognized by physicians. So, while I was excited to see a more nuanced revision to the definition of pain, I find myself waiting to see what effect this would take in the clinic office or on the hospital unit floor, if at all. Words while they can have the power to move concepts forward in medicine, like the childhood game of “Telephone”, they can also have a way of sometimes getting tripped up and skewed in intent when put into practice.

Patients and caregivers are often seen as unreliable narrators of pain.

I remember hosting a roundtable session for rare disease patients that was meant to help determine priorities in healthcare. One patient mentioned that in order to be taken seriously in the emergency department, they had to use a wheelchair that was needed on only a small handful of occasions. That stuck with me over the years as it provided context to my own experiences as a parent realizing how many times that I was probably gaslit about the severity of medical needs in tense moments. I don't know if I’d consider this slow revelation a survival tactic, but my faith in others was challenged with a great frequency. It was to a point where you had to anticipate the worst in potential outcomes from care relationships but hope for the good results.

Life demanded that my home function as a mini hospital which meant I could stay home much more often than most people. So, when we went to the emergency department, care needs were something serious and beyond the scope of my expertise. Yet, there were times in my search for support or insight that my emotions were downplayed despite my struggles to keep an intellectually disabled child in obvious, violent distress safe in the moment. “*Are you getting enough respite?*”, was a question that I found a progressive amount of distaste in as it was never about me needing a break. I would have been perfectly fine with a response of “*I don't know what's going on*” as that acknowledges the reality of how far we have yet to go in understanding the human body in its association with medical complexity. “*I don't know*”, in reality should lead to the next question, “*How can we at least make them comfortable while we figure it out*”? In some cases, we left in the same state we came in, and were offered no help.

To me, a shift in definition, while inspired in nature, requires an examination of the biases that often impede its implementation. We need to consider self‐examination and recognition of the work we still must do to address intersectional biases in gender, race, and disability. Nothing makes a patient or caregiver feel more helpless than a misaligned relationship in care that fails to examine the depth and nuance of managing one's quality of life. The stakes are so much higher with diagnoses that produce an alarming risk of suicidality.

Anton Chekhov is reputed to have said, “*Don't tell me the moon is shining, show me the glint of light on broken glass*”.^9^ While words have the capability of emotional connection and a building of trust, its impact is only truly felt with compassion, action and intent. Show us change for the better. Please.

## 3 CONCLUDING REMARKS

Pain is lived and managed in the countless moments of each day, over months, and many years. These lived experiences are charged with emotion brought, in part, by frequent encounters with the health system that leave a continuous desire for improvement. Each lived experience is unique, and these are not meant to reflect all perspectives of people who have lived with pain during childhood or cared for those who do. What these two personal accounts reflect is a skeptical hope for the future. The words captured in the revised definition of pain must be enacted with observable change and impact in culture, knowledge, and behavior.

We have had a shared language to describe pain experience since 1979, in the original IASP definition of pain.^8^ Despite this, more than 40 years later, equitable and widespread access to pain care for children remains problematic, evidence‐based pediatric pain management practices are inconsistently implemented leading to preventable and undertreated pain, and pain education for healthcare professionals remains inadequate.^3^ This suggests a pervasive lack of understanding of the definition of pain at the point of care and in the public. What use is a revised definition of pain if it does not actually benefit the people living with pain everyday? Who bears the responsibility to reach beyond the “pain community” to ensure this revised definition is put into action?

Our collective next steps require acknowledging that the work is not complete. It took more than 40 years to revise the definition of pain, and implementation cannot take so long, nor can the need to continuously review the definition over time. We must mobilize together. With the combined efforts of researchers, healthcare professionals, and policymakers, in partnership with diverse people with lived experience, we can ensure that more effective action is taken to rapidly improve pain for children and their families.

## CONFLICT OF INTEREST

The authors have no conflicts of interest to declare.

## References

[1] Belton J , Smith B . The IASP Global Alliance of Partners for Pain Advocacy (GAPPA): Incorporating the lived experience of pain into the study of pain. RELIEF: Pain Research News, Insights, and Ideas, March 18, 2020. Available at: https://relief.news/2020/03/18/the‐iasp‐global‐alliance‐of‐pain‐patient‐advocates‐gappa‐incorporating‐the‐lived‐experience‐of‐pain‐into‐the‐study‐of‐pain/

[2] Caes L , Boerner KE , Chambers CT , et al. A comprehensive bibliometric analysis of published research articles on pediatric pain from 1975–2010. Pain. 2015;157:302‐313. 10.1097/j.pain.0000000000000403 26529270

[3] Eccleston C , Fisher E , Howard RF , et al. Delivering transformative action in paediatric pain: A Lancet Child & Adolescent Health Commission. Lancet Child Adolescent Health. 2021;5:P47‐87. 10.1016/S2352-4642(20)30277-7 33064998

[4] International Association for the Study of Pain . Global Alliance of Partners for Pain Advocacy (GAPPA) Task Force. Washington, DC: International Association for the Study of Pain; 2019. Available at: https://www.iasp‐pain.org/About/Content.aspx?ItemNumber=9424

[5] Lawson JR . Letter. Birth. 1986;13:124‐125.

[6] McCaffery M . Nursing Practice Theories Related To Cognition, Bodily Pain, and Man‐environment Interactions. Los Angeles, CA: UCLA Students’ Store; 1968.

[7] McGrath PJ . Science is not enough: The modern history of pediatric pain. Pain. 2011;152:2457‐2459. 21880428 10.1016/j.pain.2011.07.018

[8] Raja SN , Carr DB , Cohen M , et al. The revised International Association for the Study of Pain definition of pain: Concepts, challenges, and compromises. Pain. 2020;161:1976‐1982. 32694387 10.1097/j.pain.0000000000001939 PMC7680716

[9] Yarmolinsky A The Unknown Chekhov: Stories and Other Writings Hitherto Untranslated by Anton Chekhov. New York, NY: Noonday Press, 1954.

